# Association Between Asthma and Obesity Among Immigrant Asian Americans, California Health Interview Survey, 2001–2011

**DOI:** 10.5888/pcd11.140333

**Published:** 2014-11-26

**Authors:** Benjamin J. Becerra, Christy M. Scroggins, Monideepa B. Becerra

**Affiliations:** Author Affiliations: Benjamin J. Becerra, Loma Linda University, Loma Linda, California; Christy M. Scroggins, California State University, San Bernardino, California.

## Abstract

Our objective was to study the comorbidity of asthma and obesity among foreign-born Asian Americans, by subgroups. Public data from the California Health Interview Survey, 2001–2011, were analyzed by using independent logistic regressions, yielding the association between asthma and obesity (Asian and standard cutoffs for body mass index [BMIs]) of 19,841 Asian American immigrant respondents. Chinese, Filipino, South Asian, and Japanese immigrants had a positive association between lifetime asthma and obesity, whereas among Korean immigrants, a positive association was found between lifetime asthma and overweight status (standard BMI cutoffs). Routine screening for this comorbidity is warranted among immigrant Asian Americans.

## Objective

Studies have demonstrated the comorbidity of asthma and obesity (1); however, few studies focus specifically on Asian Americans by subgroup. Although the prevalence of asthma among Asian Americans (8%) is lower than among whites (9%), the prevalence level varies for each Asian ethnic group with the highest (11%) reported among Chinese (2). Similarly, rates of obesity among Asian subgroups are diverse, ranging from 14% (Filipinos) to 3% (Koreans) (3). Given the limited literature on Asian Americans, our objective was to evaluate the asthma–obesity nexus among immigrant Asian American subgroups. Immigrants face unique barriers to good health (4,5); thus, such an evaluation could facilitate targeted preventive measures.

## Methods

We analyzed the publically available data on adult Asian American immigrants from the California Health Interview Survey, 2001–2011 (CHIS). CHIS uses a random-digit–dial system and is conducted in several Asian languages. A description of the CHIS design and methods can be found online (6). Only data on participants reported as 1) Asian American and 2) Asian immigrant (foreign-born) by CHIS were included in this study, resulting in a total of 19,841 respondents who represent the average annual population estimate of 2,389,910 immigrant Asian Americans in California. The Asian American subgroups included in this study were Chinese, Filipino, South Asian, Japanese, Korean, and Vietnamese. The South Asian subgroup in CHIS includes those with ancestry from Bangladesh, India, Pakistan, and Sri Lanka.

The outcome variable for the study was lifetime prevalence of asthma, defined as respondents reporting a doctor diagnosis of asthma. The independent variable was body mass index (BMI [weight in kg divided by height in m^2^]). CHIS-provided BMI was categorized on the basis of the World Health Organization’s Asian BMI ranges (7): under 23 kg/m^2^ (underweight and normal collapsed because of low underweight sample size), 23 to 27.49 kg/m^2^ (overweight), and 27.5 kg/m^2^ or above (obese). The standard BMI ranges, where 30 kg/m^2^ or higher is obese, were also assessed for comparison with Asian BMI ranges. Control variables included age, sex, marital status, socieoeconomic status, education level, medical insurance status, English language proficiency, and smoking status. Categorization of control variables was based on natural breakpoints in the distribution of participants.

Design-based *F* values were used to determine significant differences in the characteristics of each Asian American immigrant subgroup. Independent logistic regression analyses were conducted for each Asian American immigrant subgroup and each BMI categorization. Because of the survey’s complex design, a jackknife approach was used to compute standard errors. SAS 9.4 (SAS Institute, Inc) was used for all data analyses. The study was approved by the California State University, San Bernardino, Institutional Review Board.

## Results

A 7.6% lifetime prevalence of asthma was found for the study group overall, ranging from 4.9% among Koreans to 13% among Filipinos, Mean age of respondents ranged from approximately 40 years (South Asians) to 52 years (Japanese) (Table 1). Among each subgroup, with the exception of South Asians, most respondents were female. Most Asian American immigrants in each subgroup were married, had a bachelor’s degree or higher (with the exception of Japanese and Vietnamese), lived at or above the 200% federal poverty level (except Vietnamese), and had medical insurance during all past 12 months. The highest prevalence of obesity (based on Asian and standard BMI cutoffs) and asthma was among Filipinos, followed by South Asians.

**Table 1:** Sociodemographic and Other Characteristics, Study of Association Between Asthma and Obesity Among Immigrant Asian Americans^a^ (N = 19,841)^b^, California Health Interview Survey, 2001–2011

Variables^c^	Chinese (n = 5,782)	Filipino (n = 2,935)	South Asian (n = 2,288)	Japanese (n = 652)	Korean (n = 3,827)	Vietnamese (n = 4,357)
**Age, mean (SE)**	46.17 (±0.27)	47.53 (±0.36)	39.45( ±0.36)	51.65( ±0.92)	46.44 (±0.42)	45.04 (±0.35)
**Sex, n (%)**						
Male	2,385 (45.0)	1,087 (42.6)	1,216 (56.9)	195 (29.8)	1,404 (40.4)	2,137 (49.3)
Female	3,397 (55.0)	1,848 (57.4)	1,072 (43.1)	457 (70.2)	2,423 (59.3)	2,220 (50.7)
**Marital status, n (%)**						
Currently married	4,050 (69.7)	1,907 (65.4)	1,814 (77.7)	377 (65.5)	2,676 (69.0)	2,973 (66.1)
Currently not married	1,732 (30.3)	1,028 (34.6)	474 (22.3)	275 (34.5)	1,151 (31.0)	1,384 (33.9)
**Educational status, n (%)**						
Associate degree or less	2,584 (48.4)	1,161 (44.6)	439 (19.9)	315 (50.3)	1,750 (44.5)	3,145 (72.3)
Bachelor’s degree or more	3,198 (51.6)	1,774 (55.4)	1,849 (80.1)	337 (49.7)	2,077 (55.5)	1,212 (27.7)
**Socioeconomic level, n (%)**						
Living at or above 200% FPL	3,775 (65.1)	2,187 (74.0)	1,919 (83.3)	522 (80.1)	2,256 (65.4)	1,791 (44.4)
Living below 200% FPL	2,007 (34.9)	748 (26.0)	369 (16.7)	130 (19.9)	1,571 (34.6)	2,566 (55.6)
**Insured all past 12 months, n (%)**						
Yes	4,855 (83.1)	2,579 (86.0)	2,011 (86.8)	575 (88.4)	2,635 (59.6)	3,568 (77.7)
No	927 (16.9)	356 (14.0)	277 (13.2)	77 (11.6)	1192 (40.4)	789 (22.3)
**Smoking status, n (%)**						
Ever smoker	1,157 (21.0)	874 (29.9)	376 (16.3)	252 (36.9)	1,243 (36.9)	1,307 (29.6)
Never smoker	4,625 (79.0)	2,061 (70.1)	1,912 (83.7)	400 (63.1)	2,584 (63.1)	3,050 (70.4)
**English language proficiency, n (%)**						
Not English proficient	2,279 (39.9)	189 (5.9)	74 (3.1)	124 (19.3)	2,221 (53.0)	2,551 (53.4)
English proficient	3,503 (60.1)	2,746 (94.1)	2,214 (96.9)	528 (80.68)	1,606 (47.0)	1,806 (46.6)
**Asian BMI (kg/m^2^), n (%)**						
Less than 23.00	3,098 (53.8)	1,026 (33.2)	862 (39.8)	372 (56.9)	1,919 (51.3)	2,310 (60.0)
23.00–27.49	2,162 (37.7)	1,318 (46.3)	1,067 (45.0)	214 (32.1)	1,589 (40.4)	1,506 (30.2)
27.50 or higher	522 (8.5)	591 (20.6)	359 (15.9)	66 (11.0)	319 (8.3)	541 (9.8)
**Standard BMI (kg/m^2^), n (%)**						
Less than 25.00	4,375 (76.3)	1,684 (55.0)	1,439 (64.1)	490 (74.3)	2,828 (73.6)	3,217 (77.7)
25.00–29.99	1,187 (20.2)	998 (36.1)	684 (29.2)	125 (20.9)	897 (24.0)	815 (16.9)
30.00 or more	220 (3.6)	253 (8.9)	165 (6.7)	37 (4.8)	102 (2.4)	325 (5.4)
**Lifetime asthma prevalence, n (%)**	320 (5.2)	342 (13.0)	179 (7.8)	46 (6.6)	212 (4.9)	300 (5.3)

Abbreviations: SE, standard error; FPL, federal poverty level; BMI, body mass index.

a The California Health Interview Survey defines South-Asian Americans as those from Bangladesh, India, Pakistan, and Sri Lanka.

b Total California Asian American population estimate from the California Health Interview Survey, 2001–2011, was 2,389,910.

c Percentages may not add to 100% because of rounding.

Results from unadjusted regression analyses (data not shown) demonstrate significant associations between lifetime prevalence of asthma and obesity among Chinese, Filipinos, and South Asians, regardless of the BMI cutoffs used. Among Japanese and Korean subgroups, respondents who were overweight were more likely to have lifetime asthma according to standard BMI cutoffs. After adjusting for control variables (age, sex, marital status, socioeconomic level, education, medical insurance status, English language proficiency, and smoking status), the association between asthma and obesity varied by Asian American subgroup. The adjusted odds of asthma among those with BMI of 27.5 kg/m^2^ or more (considered obese by Asian BMI cutoffs) ranged from 1.92 (95% confidence interval, 1.18–3.10) among Chinese to 3.34 (95% CI, 1.57–7.93) among Japanese. A positive relationship between lifetime prevalence of asthma and obesity according to Asian and standard BMI cutoffs remained for Chinese, Filipinos, and South Asians (Table 2). Among Japanese, asthma was associated with overweight status by both Asian and standard BMI cutoffs and with obesity by Asian BMI cutoffs. However, among Koreans, asthma was associated with only overweight status based on standard BMI cutoffs. 

**Table 2:** Multivariable^a^ Logistic Regression Analysis of Lifetime Asthma Among Immigrant Asian Americans^b^, by BMI Categories for Asians and Non-Asians, California Health Interview Survey, 2001–2011

Asian American Subgroup	BMI (kg/m^2^)		
	OR (95% CI)	OR (95% CI)	OR (95% CI)
**Model based on standard BMI categories^c^**	**<25**	**25–29.9**	**≥30**
Chinese	1 [Reference]	0.96 (0.63–1.46)	1.89 (1.04–3.44)^d^
Filipino		1.25 (0.85–1.85)	2.76 (1.72–4.43)^e^
South Asian		1.55 (0.92–2.63)	2.47 (1.39–4.40)^e^
Japanese		3.13 (1.39–7.07)^e^	3.15 (0.71–13.94)
Korean		1.83 (1.09–3.08)^d^	1.20 (0.49–2.96)
Vietnamese		1.16 (0.70–1.90)	1.54 (0.82–2.91)
**Model based on Asian-specific BMI categories^f^**	**<23**	**23–27.5**	**≥27.5**
Chinese	1 [Reference]	1.27 (0.86–1.86)	1.92 (1.18–3.10)^e^
Filipino		1.41 (0.93–2.13)	2.13 (1.33–3.42)^e^
South Asian		0.69 (0.44–1.09)	2.32 (1.37–3.95)^e^
Japanese		2.23 (1.24–4.02)^e^	3.34 (1.57–7.13)^e^
Korean		1.30 (0.82–2.06)	1.64 (0.78–3.45)
Vietnamese		1.07 (0.70–1.63)	1.45 (0.82–2.58)

Abbreviations: BMI, body mass index; OR, odds ratio; CI, confidence interval.

a Multivariable logistic regression model adjusted for age, sex, marital status, poverty, education, insurance status, English language proficiency, and smoking status.

b The South Asian subgroup in the California Health Interview Survey includes those with ancestry from Bangladesh, India, Pakistan, and Sri Lanka.

c Categorized based on standard BMI cutoffs (http://www.cdc.gov/healthyweight/assessing/bmi/adult_bmi/index.html).

d *P* <.05.

e *P <* .01.

f Categorized based on the World Health Organization’s Asian BMI cutoffs (7): under 23 kg/m^2^, underweight and normal (underweight and normal collapsed because of low underweight sample size); 23 to 27.49 kg/m^2^, overweight; and at or above 27.5 kg/m^2^, obese.

### Discussion

Lifetime prevalence of asthma was diverse in our Asian American immigrant population, with Filipinos reporting the highest. Such results further demonstrate the diversity among the heterogeneous Asian American population. Although we found that the overall asthma prevalence was lower for most Asian American immigrant subgroups in our study than the prevalence for all Californians as reported by the California Department of Public Health (8), the significant association between asthma and obesity warrants routine preventive screening among Asian Americans to lower the burden of the comorbidity. The results found in our study among South Asians are consistent with previous research conducted in India (9), indicating the burden of the asthma–obesity nexus in the population, irrespective of country of residence. Given that recent studies have shown limited knowledge of asthma among Asian Americans (10), public health efforts to improve asthma literacy in this population should be a priority. The relationship between asthma and overweight status among Koreans (using standard BMI cutoffs) further shows the heterogeneity among Asian Americans and the need for clinicians to use both standard and Asian BMIs when assessing risk status.

Although results of our study did not reach statistical significance for modeling the relationship between asthma and obesity among Vietnamese immigrants, a recent publication notes the increasing rates of overweight status in Vietnam (11). Therefore, future studies should evaluate the relationship between asthma and obesity, because the Vietnamese immigrant population continues to grow in the United States (12). 

The self-reported data in CHIS is susceptible to recall bias, and given that CHIS is California-based, our results are not generalizable to other states. Despite such limitations, the use of a population-based survey allows results to be generalizable to the Asian American immigrant population in California. The findings from this study highlight the need for public health efforts to address the asthma–obesity nexus among Asian Americans overall and to reach specific Asian American subgroups to prevent this comorbidity.
